# Supplementary material for: Confidence-weighted integration of human and machine judgments for superior decision-making
Source: Patterns (N Y). 2025 Nov 20;7(2):101423. doi: 10.1016/j.patter.2025.101423 (PMC12921503; doi:10.1016/j.patter.2025.101423)
Supplement: Document S1. Figures S1–S8 and supplemental methods [file mmc1.pdf]

**Patterns, Volume *7***

## Supplemental information

# Confidence-weighted integration of human and machine judgments for superior decision-making

**Felipe Yáñez, Xiaoliang Luo, Omar Valerio Minero, and Bradley C. Love**

# SUPPLEMENTAL FIGURES

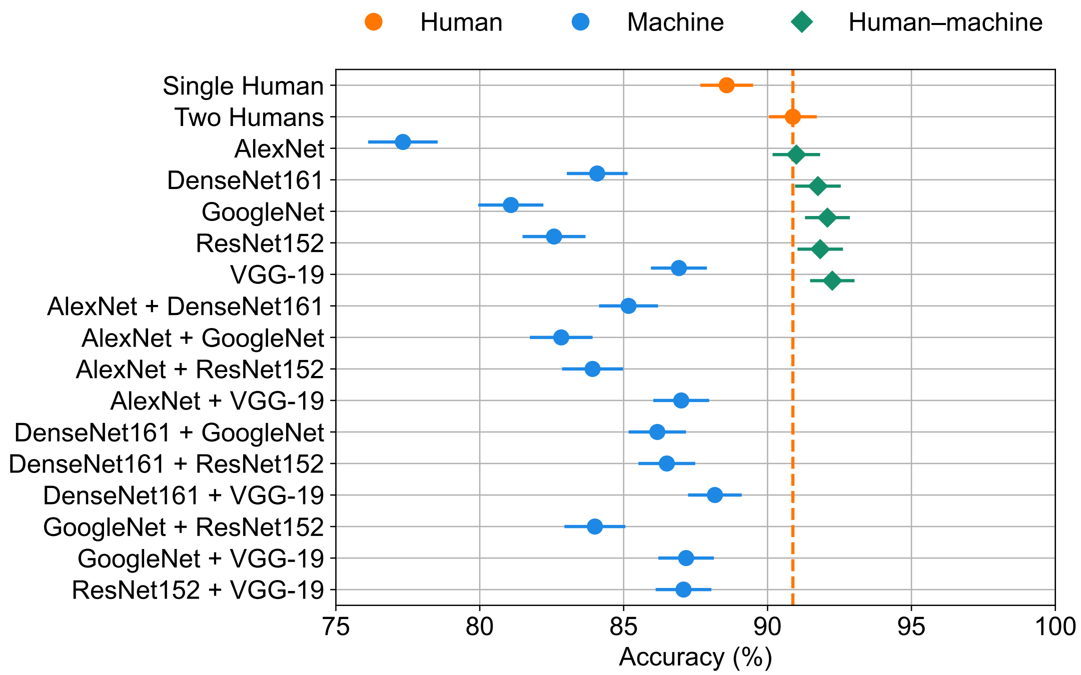


**Figure S1. Performance of the Bayesian combination models in the low-noise object recognition task [S1]**

We reproduced Steyver's et al.'s [S1] results using ordered logit (see Equation 2), as opposed to probit. Accuracy results on low levels of image noise ($\Omega = 80$) with the Bayesian combination model, reproducing the original setting. We used 1200 predictions (corresponding to 1200 unique images) and split them into four random partitions for the purpose of four-fold cross-validation. Two human participants were created by random sampling without replacement across the 1200 unique images. In this case, machine classifiers are surpassed by humans ($t(4) = -3.88$, $P < 0.01$). Teams comprising a human and a machine (green points) deliver superior results compared to machine-only and machine–machine teams (Welch's $t(16.04) = 9.18$, $P < 0.0001$). Each data point corresponds to the average across 1200 image evaluations. Error bars represent standard error of the mean using a binomial model.

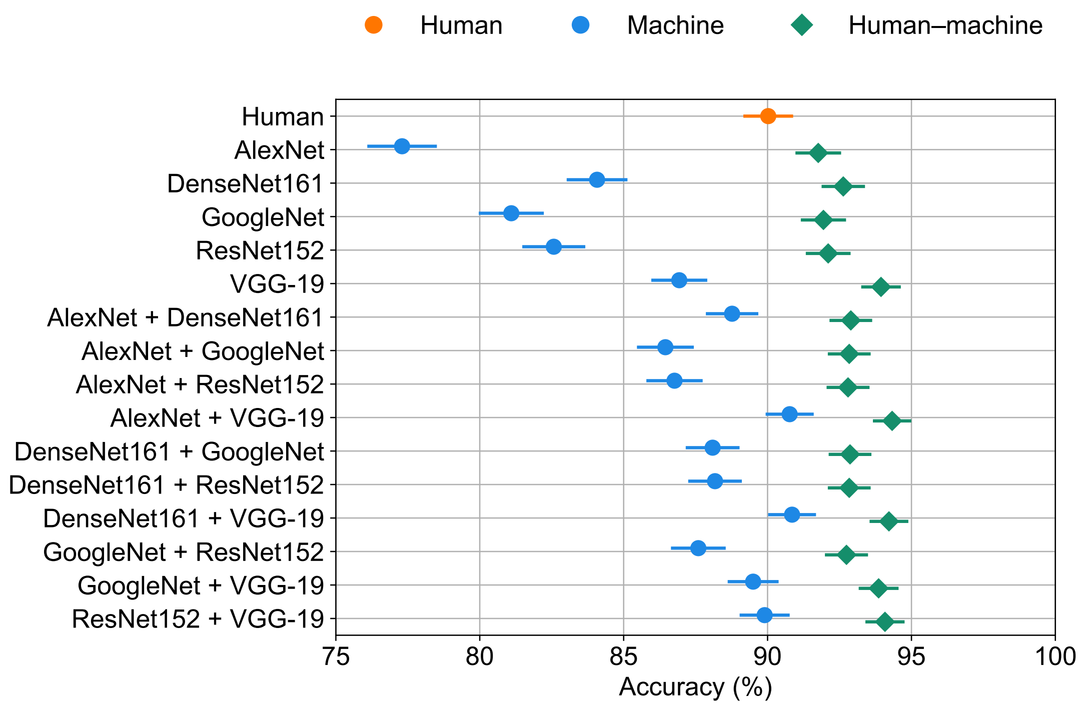


## Figure S2. Performance of the confidence-weighted logistic combination model in the low-noise object recognition task [S1]

Accuracy results on low levels of image noise ($\Omega = 80$) with the confidence-weighted combination model, where humans outperform machines ($t(4) = -4.77$, $P < 0.01$). Human–machine teams consistently outperform teams with one or more machines (Welch's $t(37.46) = 12.50$, $P < 0.0001$). Each data point corresponds to the average across 7247 image evaluations. Error bars represent standard error of the mean using a binomial model.

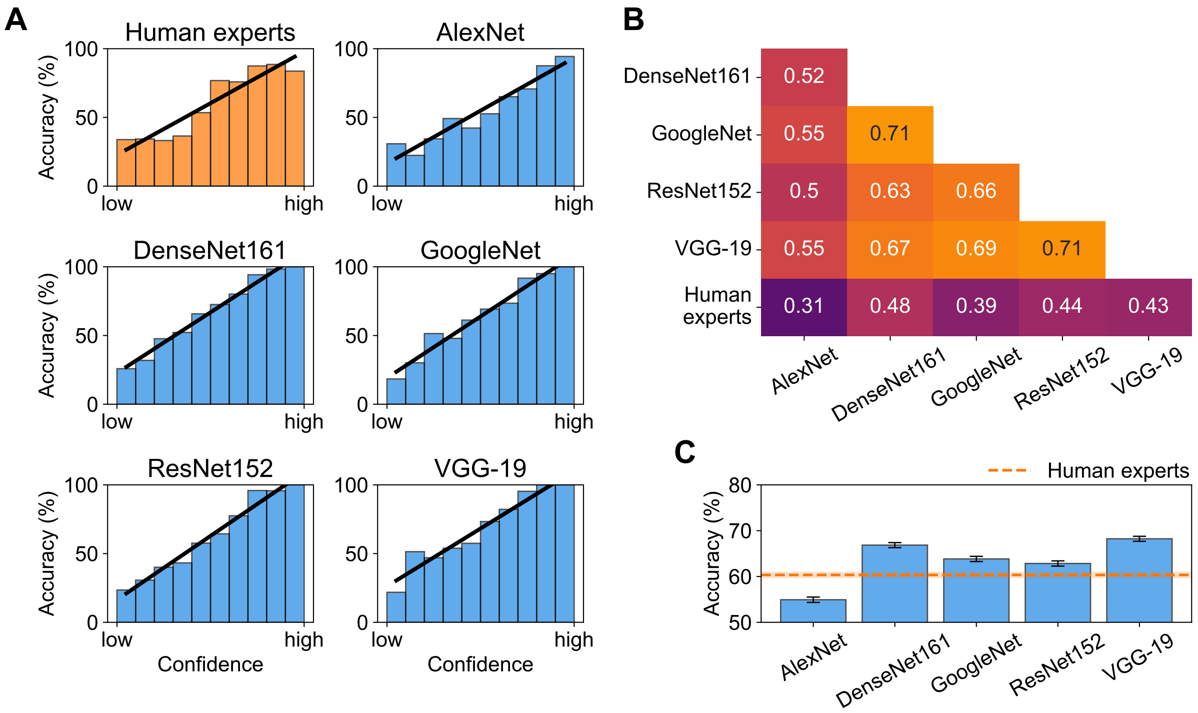


**Figure S3. Conditions for effective collaboration between human experts and machines are satisfied for the noisy object recognition task [S1]**

(A) When human experts and machines were confident in their judgments, they were more likely to be correct. Confidence ratings were sorted into equal bins, and the mean accuracy for each bin was plotted. The positive slope of the black regression lines for humans and models (AlexNet, DenseNet161, GoogleNet, ResNet152, and VGG-19) indicates well-calibrated confidence [S2–S5], meaning higher confidence correlates with higher accuracy.

(B) Item difficulty Spearman correlations among machines and human experts. For machines, we used the probability score of the predicted image class to determine relative image classification difficulty. The probability score was then signed with $+1$ if the prediction was correct and $-1$ if it was incorrect. Mean accuracy was used for human experts. Heatmap color scale ranges from 0.1 to 0.9.

(C) Besides AlexNet, models surpass human experts on this task. Error bars represent standard error of the mean using a binomial model.

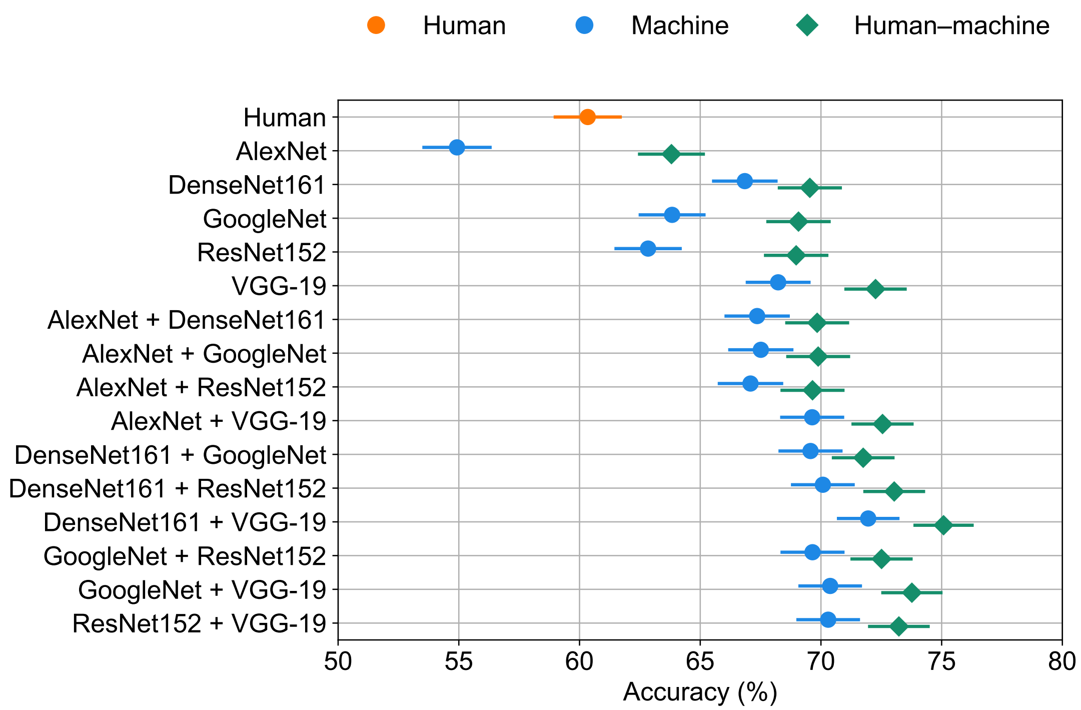


**Figure S4. Removing confidence from the confidence-weighted logistic combination model does not negatively impact team performance in the noisy object recognition task [S1]**

Accuracy results on high levels of image noise ($\Omega = 125$) with the confidence-weighted logistic combination model, where the probability scores were set to 1, i.e., $f(x) = 1$ in Equation 4. Similarly as in the base scenario (Figure 1), human–machine teams surpass machine-only teams (Welch's $t(24.08) = 2.80$, $P < 0.01$), although with a smaller margin. Each data point corresponds to the average across 7239 image evaluations. Error bars represent standard error of the mean using a binomial model.

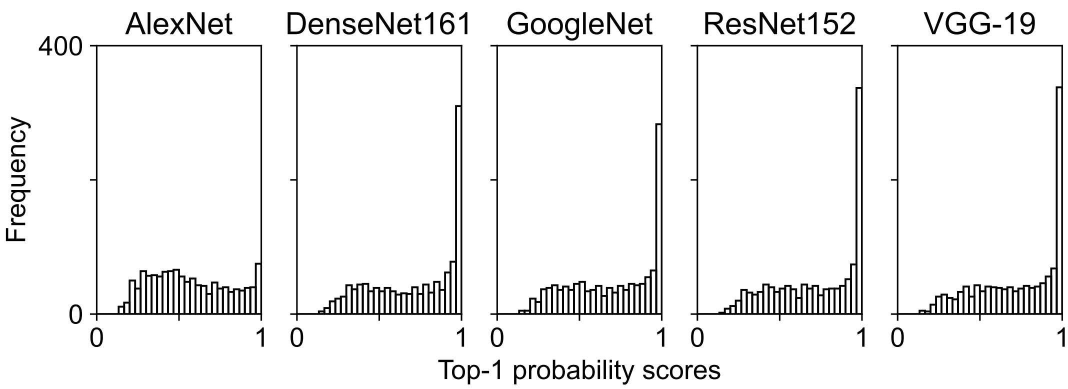


## Figure S5. Confidence assessment in the noisy object recognition task [S1]

Histogram of top-1 probability scores under high image noise ($\Omega = 125$) for different machine classifiers. For each test case ($n = 1200$), only the highest predicted probability was used, and values were grouped into 30 bins. The resulting distributions are heavily skewed toward 1. To test whether confidence magnitudes improve predictions, we used cross-entropy, a metric that reflects both accuracy and calibration. For each classifier, cross-entropy was computed independently for both signed probability scores (i.e., signed confidence) and sign-only features. The results are nearly identical (Welch's $t(7.99) = 0.24$, $P = 0.82$), suggesting that confidence magnitudes provide no additional benefit in this setting.

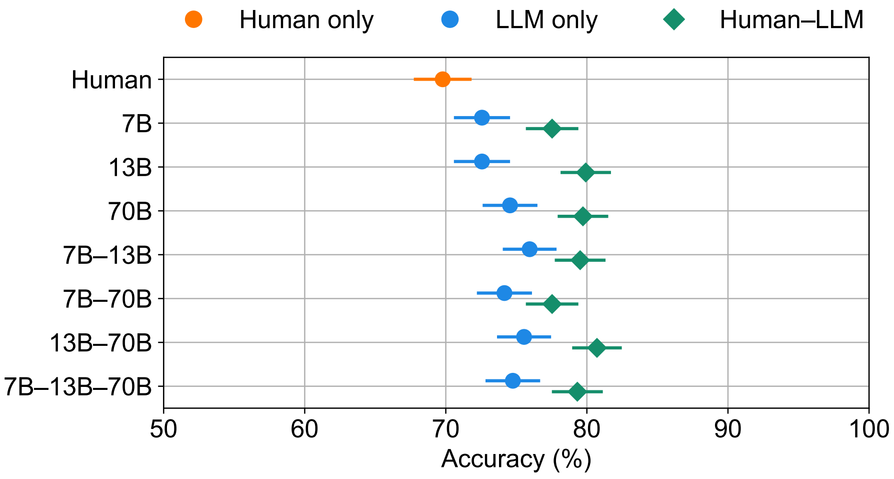


**Figure S6. Optimal squashing in the confidence-weighted logistic combination model does not improve overall team performance in the neuroscience forecasting task [S5]**

Accuracy results on the neuroscience forecasting task with the confidence-weighted logistic combination model, where the parameter $\alpha$ in Equation 4 was optimized. Similarly as in the base scenario (Figure 4), human–LLM teams surpass LLM-only teams (Welch's $t(11.89) = 7.20$, $P < 0.0001$). The performance between base and squashing scenarios is, however, indistinguishable (Welch's $t(19.32) = 0.05$, $P = 0.96$). Each data point corresponds to the average across 503 test case evaluations. Error bars represent standard error of the mean using a binomial model.

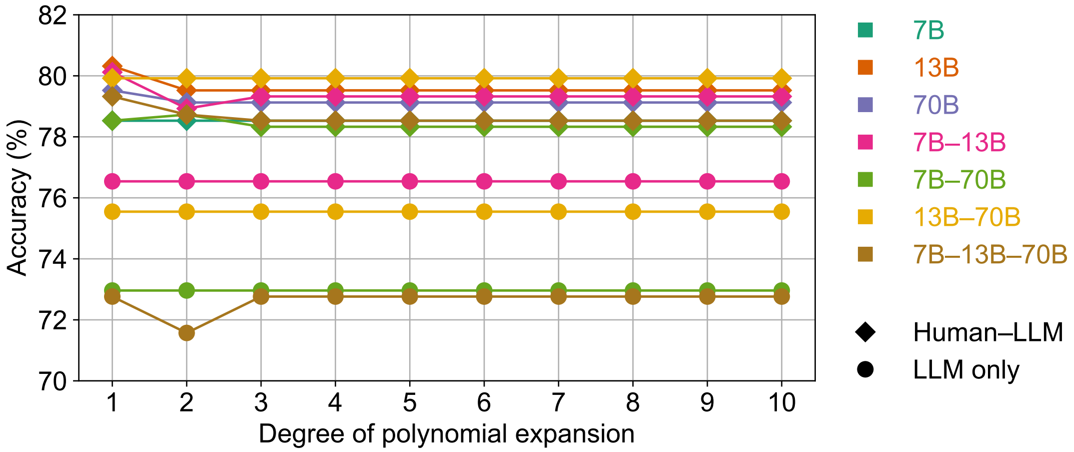


**Figure S7. Adding interaction terms to the confidence-weighted logistic combination model does not improve overall team performance in the neuroscience forecasting task [S5]**

Accuracy results of the confidence-weighted logistic combination model on the neuroscience forecasting task as a function of the degree of the polynomial expansion of the features. Only interaction terms were considered in the polynomial expansion, as the results including pure terms were identical. Team performance is independent of interaction terms. Each data point corresponds to the average across 503 test case evaluations.

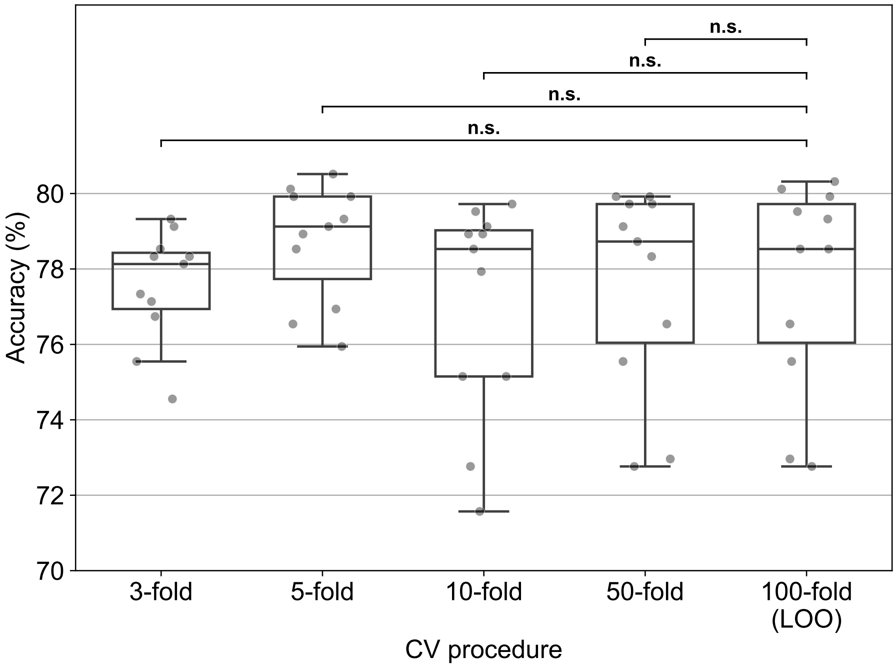


**Figure S8. Performance consistency analysis across different cross-validation procedures**

Box plots show the distribution of overall accuracy for 3-fold, 5-fold, 10-fold, 50-fold, and leave-one-out (LOO) cross-validation (CV) procedures for the experiment presented in Figure 4. Overlaid data points represent human–machine and machine–machine team predictions. Paired t-tests (parametric) and Wilcoxon signed-rank tests (non-parametric) were used to compare each CV procedure with LOOCV to ensure robust assessment regardless of data distribution assumptions. Results show non-significant differences, with similar mean accuracies (ranging from 77.0% to 78.7%) and overlapping 95% bootstrap confidence intervals (widths between 1.8% and 3.2%). This demonstrates that LOOCV is an appropriate validation procedure to evaluate model performance.

# SUPPLEMENTAL METHODS

## Implementation of Bayesian Combination Model

We implemented the Bayesian combination model [S1] in Python [S6], which was originally developed in JAGS. The model comprises two stages: parameter inference (training) and class label prediction (testing). Algorithm S1 illustrates the inference procedure for the human–machine case. During testing, the posterior samples of the parameters learned in training are used to evaluate unseen data. For each posterior sample, we compute the joint log-likelihood of the machine scores, human classifications, and human confidence ratings under each possible class. These log-likelihoods are aggregated across samples and normalized via a softmax transformation. The predicted class label corresponds to the class with the highest probability.

In Figure S1, we reproduced the experiment from Figure 3 (Top) in Steyver's et al. [S1] using our Python implementation. The overall team performance ($n = 16$) obtained with our method is indistinguishable from the original JAGS implementation (Welch's $t(28.95) = -0.24$, $P = 0.81$). This also holds for the five human–machine (Welch's $t(5.33) = -2.17$, $P = 0.08$), and ten machine–machine (Welch's $t(17.60) = 0.17$, $P = 0.87$) teams.

---

**Algorithm S1** Bayesian Combination Model

---

Given data: true class labels $z$, probability scores $\pi_M$, human classification $y$, and confidence ratings $r$.

Set priors: $a_M \sim \mathcal{N}(0, 10)$, $b_M \sim \mathcal{N}(0, 10)$, $\sigma_M \sim$ Uniform$(0, 15)$, $a_H \sim \mathcal{N}(0, 10)$, $b_H = 0$, $\sigma_H = 1$, $\rho \sim$ Uniform$(-1, 1)$, $\tau = 0.05$, $c \sim$ Uniform$(0, 1)$ with $c_i < c_{i+1}\ \forall\, i = 1, \ldots, R-2$, and $\delta \sim$ Uniform$(0, 100)$.

**start parameter inference:**

  $\mu_{i,j,M} \leftarrow b_M + (a_M - b_M) \cdot \mathbf{1}_{\mathcal{Z}_i}(j)$

  $\mu_{i,j,H} \leftarrow b_H + (a_H - b_H) \cdot \mathbf{1}_{\mathcal{Z}_i}(j)$

  $\pi_M \sim \mathcal{N}\left(\mu_M, \sigma_M\right)$ ▷ Compare to actual data

  $\pi_H \sim \mathcal{N}\left(\mu_H + \rho\,\sigma_H \left(\frac{\pi_M - \mu_M}{\sigma_M}\right), \sqrt{1-\rho^2}\,\sigma_H\right)$

  $y \sim$ Categorical (Softmax $(\pi_H/\tau)$) ▷ Compare to actual data

  $r \sim$ OrderedLogistic$(\pi_H, c, \delta)$ ▷ Compare to actual data

**end parameter inference**

---

We assumed that all human participants shared the same set of parameters ($a_H$ , $b_H$ , $\sigma_H$, $c$, $\delta$, and $\tau$). In the neuroscience forecasting task, human confidence ratings on the slider bar were mapped to range between 1 and 100. A wide range is computationally expensive, thus, we aggregate it into three levels: "0: low confidence", "1: moderate confidence", and "2: high confidence". Then, the aggregated confidence rating used for analysis, $r \in \{0, 1, 2\}^N$, reads

$$r = \begin{cases} 0 & \text{if} \qquad\quad \text{self-reported confidence} \leq 33, \\ 1 & \text{if}\ \ 33 < \text{self-reported confidence} \leq 66, \\ 2 & \text{if}\ \ 66 < \text{self-reported confidence} \end{cases} .$$

The utilized cutpoints (i.e., 33 and 66) produced a good agreement between confidence and accuracy. Among the evaluations of human participants, "low" had $63.2\%$ average accuracy ($n = 174$), "moderate" had $66.5\%$ ($n = 185$), and "high" had $81.9\%$ ($n = 144$). To infer the posterior over the underlying parameters, a No-U-Turn Sampler (NUTS) for Markov chain Monte Carlo (MCMC) [S7] was used with $n_w = 1000$ warmup steps, $n_c = 8$ chains, and $n_s = 50$ samples.

## SUPPLEMENTAL REFERENCES


[S1] Steyvers, M., Tejeda, H., Kerrigan, G., and Smyth, P. (2022). Bayesian modeling of human–AI complementarity. PNAS *119*, e2111547119. https://doi.org/10.1073/pnas.2111547119.

[S2] Keren, G. (1991). Calibration and probability judgements: Conceptual and methodological issues. Acta Psychol. *77*, 217–273. https://doi.org/10.1016/0001-6918(91)90036-Y.

[S3] Baranski, J. V. and Petrusic, W. M. (1994). The calibration and resolution of confidence in perceptual judgments. Percept. Psychophys. *55*, 412–428. https://doi.org/10.3758/BF03205299.

[S4] Tian, K., Mitchell, E., Zhou, A., Sharma, A., Rafailov, R., Yao, H., Finn, C., and Manning, C. (2023). Just Ask for Calibration: Strategies for Eliciting Calibrated Confidence Scores from Language Models Fine-Tuned with Human Feedback. In Proceedings of the 2023 Conference on Empirical Methods in Natural Language Processing, H. Bouamor, J. Pino, and K. Bali, eds. (Association for Computational Linguistics), pp. 5433–5442. https://doi.org/10.18653/v1/2023.emnlp-main.330.

[S5] Luo, X., Rechardt, A., Sun, G., Nejad, K. K., Yáñez, F., Yilmaz, B., Lee, K., Cohen, A. O., Borghesani, V., Pashkov, A., et al. (2025). Large language models surpass human experts in predicting neuroscience results. Nat. Hum. Behav. *9*, 305–315. https://doi.org/10.1038/s41562-024-02046-9.

[S6] Yáñez, F., Luo, X., Valerio Minero, O., and Love, B. C. (2025). Source code for "Confidence-weighted integration of human and machine judgments for superior decision-making". Edmond. https://doi.org/10.17617/3.IGVPQV.

[S7] Bingham, E., Chen, J. P., Jankowiak, M., Obermeyer, F., Pradhan, N., Karaletsos, T., Singh, R., Szerlip, P., Horsfall, P., and Goodman, N. D. (2019). Pyro: Deep Universal Probabilistic Programming. J. Mach. Learn. Res. *20*, 1–6. http://jmlr.org/papers/v20/18-403.html.
