# Supplementary material for: Confidence-weighted integration of human and machine judgments for superior decision-making
Source: Patterns (N Y). 2025 Nov 20;7(2):101423. doi: 10.1016/j.patter.2025.101423 (PMC12921503; doi:10.1016/j.patter.2025.101423)
Supplement: Document S2. Article plus supplemental information [file mmc2.pdf]

Patterns

Article

Confidence-weighted integration of human and machine judgments for superior decision-making

Graphical abstract

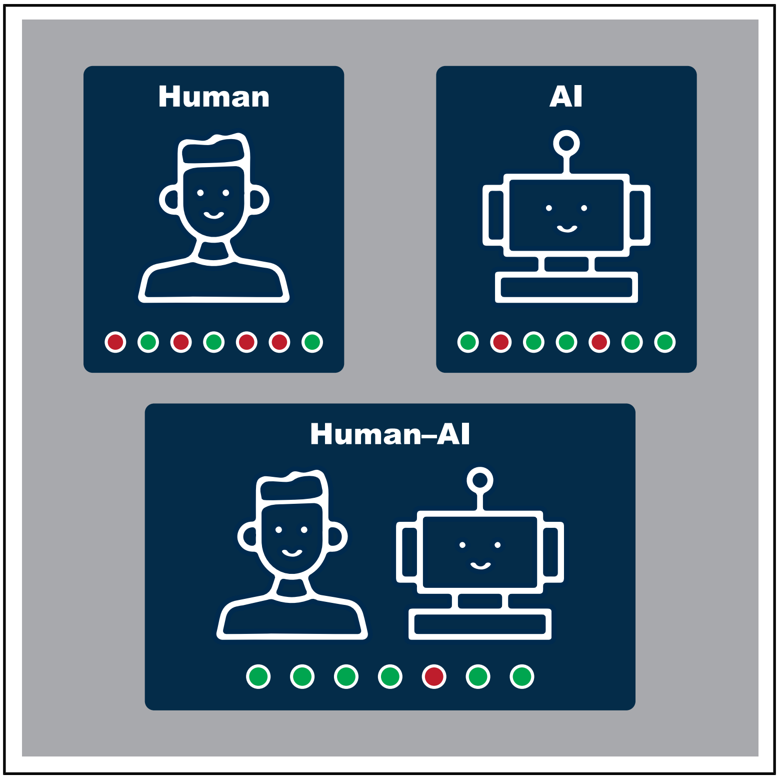

Authors

Felipe Yáñez, Xiaoliang Luo, Omar Valerio Minero, Bradley C. Love

Correspondence

felipe.yanez@mpinb.mpg.de

In brief

When AI surpasses human performance, what can humans offer? We demonstrate that the performance of teams increases by integrating human judgments with those of machines. Integration is achieved by a straightforward regression approach that combines team members' confidence-weighted judgments.

Highlights

- Requirements for effective human-AI teaming, even when AI exceeds human capabilities
- Scalable approach combining confidence-weighted judgments from humans and AI
- Human-AI teaming surpassed individual AI systems in two different forecasting tasks
- Adding a human to a team with one or more AI systems improved team performance

Yáñez et al., 2026, Patterns 7, 101423
February 13, 2026 © 2025 The Author(s). Published by Elsevier Inc.
https://doi.org/10.1016/j.patter.2025.101423

CellPress

Patterns

CellPress
OPEN ACCESS

Article

Confidence-weighted integration of human and machine judgments for superior decision-making

Felipe Yáñez,[1,4,*] Xiaoliang Luo,[2] Omar Valerio Minero,[1] and Bradley C. Love[3]
[1]Max Planck Institute for Neurobiology of Behavior – caesar, Bonn, Germany
[2]Department of Experimental Psychology, University College London, London, UK
[3]Los Alamos National Laboratory, Los Alamos, NM, USA
[4]Lead contact
*Correspondence: felipe.yanez@mpinb.mpg.de
https://doi.org/10.1016/j.patter.2025.101423

THE BIGGER PICTURE Artificial intelligence (AI) systems, such as large language models (LLMs), have emerged as powerful tools in various domains. Recent studies have shown that LLMs can surpass humans in certain tasks, such as predicting the outcomes of neuroscience studies. This raises a critical question: as AI systems achieve superhuman performance in specific domains, will they displace human judgment in critical decision-making processes? Our study shows that humans still have a lot to offer. Because humans tend to make different mistakes than machines and can express how confident they are in their decisions, human judgments can be combined with those of AI systems to form teams that are more effective than teams consisting of machines alone. At least in the near term, human judgments offer a valuable and complementary signal that can increase decision-making performance in human-machine teams or ensembles.

SUMMARY

Large language models (LLMs) can surpass humans in certain forecasting tasks. What role does this leave for humans in the overall decision process? One possibility is that humans, despite performing worse than LLMs, can still add value when teamed with them. A human and machine team can surpass each individual teammate when team members' confidence is well calibrated and team members diverge in which tasks they find difficult (i.e., calibration and diversity are needed). We simplified and extended a Bayesian approach to combining judgments using a logistic regression framework that integrates confidence-weighted judgments for any number of team members. Using this straightforward method, we demonstrated its effectiveness in both image classification and neuroscience forecasting tasks. Combining human judgments with one or more machines consistently improved overall team performance. Our hope is that this simple and effective strategy for integrating the judgments of humans and machines will lead to productive collaborations.

INTRODUCTION

Modern environments increasingly stretch our ability to process the vast amounts of information available to us.[1,2] In contrast, machine systems can often take advantage of vast information resources.[3–6] As machines reach superhuman performance levels,[5,7,8] one concern is whether machines will supplant human judgment in critical areas.[9,10]

One potential solution is forming human-machine teams in which judgments from humans and machines are integrated.[11–13] It might be possible that humans can contribute to and make the overall team better even when their performance is worse on average than their machine teammates.

We will begin evaluating this possibility in an object recognition task where human and machine performance overlap according to experimental conditions.[13] Human-machine teaming combines the individual judgments of humans and machines. We will then evaluate a knowledge-intensive task in which large language models (LLMs) surpass humans in predicting the outcomes of neuroscience studies,[14] posing a real challenge for effective team collaboration. Complementarity is realized when a team's performance improves beyond that of either teammate alone.[12,13] We investigate whether human-LLM teams outperform LLMs even when humans have inferior performance compared to LLMs. There are two key conditions for team complementarity to be fulfilled.[13–15] The first requirement is

This is an open access article under the CC BY license (http://creativecommons.org/licenses/by/4.0/).

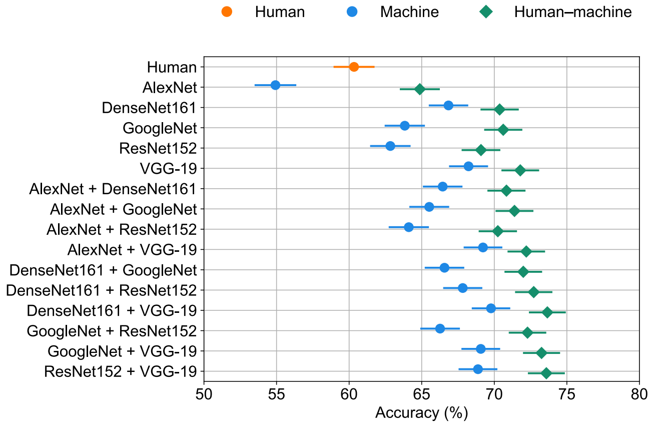

Figure 1. Performance of the confidence-weighted logistic combination model in the noisy object recognition task

Accuracy results on high levels of image noise (Ω = 125) with the logistic combination model. Human-machine teams (green points) consistently outperform teams without humans (blue points). Each data point corresponds to the average across 7,239 image evaluations. Error bars represent the standard error of the mean using a binomial model.

calibration of confidence. This implies that when humans and LLMs have a higher degree of confidence in their judgments, the accuracy of those judgments tends to be greater.[14] The second requirement is classification diversity among team members. Diversity holds when the errors in classification made by humans and LLMs are not the same.[14]

Previous work[13] has explored the conditions for complementarity in the context of object recognition. Humans outperformed machines in the classification of natural images with low levels of noise, raising the question of whether a combined approach could achieve superhuman performance. They developed a Bayesian model that integrates the judgments of humans and machines. With this approach, human-machine complementarity was observed. However, the combination model is computationally expensive and challenging to extend to additional teammates. Ideally, a model that combines the judgments of humans and machines should be adaptable and scalable, easily interpretable, and allow for any number of teammates.

Here, we aim to offer this ideal solution to human-machine teaming while evaluating complementarity in an object recognition task and a knowledge-intensive task that is not based on perceptual judgment. Critically, in both scenarios, humans were surpassed by machine systems. Foreshadowing our results, we find support for effective human-machine teaming through our resource-efficient procedure. Our procedure comprises a logistic-regression-based strategy that provides confidence-weighted integration of teammates' predictions for any number of team members. Our approach is particularly well suited for combining human and machine judgments and assessing their contribution in predictive tasks.

RESULTS

In this study, we explore whether humans can contribute to decisions when machine models, such as LLMs, are superior to them. We developed a logistic-regression-based method that integrates a weighted average of judgments from teammates, whether humans or machines. The proposed approach adheres to similar previously reported principles,[13] offering a number of advantages: it is easy to use, flexible, and resource efficient (details can be found in the methods). We evaluate our method in an object recognition task where effective human-machine collaboration has been demonstrated.[13] We then shift our focus to BrainBench[14] because LLMs significantly outperform human experts, posing a real challenge for team collaboration.

Performance is improved when a human collaborates in a machine-only team

We first assessed the performance of human-machine teams in the classification of noisy natural images.[13] The images were distorted by phase noise at each spatial frequency, where the phase noise was uniformly distributed in the interval $[-\Omega,\Omega]$.[16] We considered two noise levels: images distorted by low (Ω = 80) and high (Ω = 125) noise. In the case of low noise, machines are surpassed by humans ($t(4) = -4.77$, $p < 0.01$). The Bayesian combination model[13] demonstrated human-machine complementarity (Figure S1). Despite its simpler setup, our confidence-weighted logistic combination model was able to provide team complementarity not only for human-machine teams but also for machine-machine teams (Figure S2). Furthermore, our approach outperformed the Bayesian combination model (Welch's $t(20.87) = 2.91$, $p < 0.01$). Figure 1 shows the performance of our confidence-weighted logistic combination model in the case of high noise, where most machines outperform humans (Figure S3). Of primary interest was whether teams including humans performed better than machine-only teams. We assessed machine-only teams comprising either one or two machines. Adding a human teammate to machine-only teams always improved the team's performance (Welch's $t(22.78) = 4.70$, $p < 0.0001$). Bayesian integration is marked by combining judgments based on confidence ratings from team members, whether human or machine. Our confidence model can use this information as well. However, it remains unclear whether confidence weighting is crucial or if improved team performance simply reflects an averaging or wisdom-of-the-crowd effect. This question has not been asked with this dataset but is straightforward to evaluate using variations of our regression approach. We found that removing confidence in our approach did not negatively impact team performance in this noisy object recognition task (Figure S4). For human-machine teams, there is no difference between our standard model and the variant that does not weight by confidence (Welch's $t(26.71) = 0.29$, $p = 0.77$). Surprisingly, for machine-machine teams, the no-confidence model outperformed the standard (Welch's $t(17.47) = 2.58$, $p < 0.01$). A similar performance between signed confidence and sign-only features indicates that confidence does not contribute to improved predictions. This is a consequence of machine classifiers being skewed toward high probability scores (Figure S5). On the one hand, these results demonstrate the versatility of our approach in that different variations of our model can assess the basis for the success of human-machine teams. On the other hand, the fact that the object recognition dataset did not require confidence-weighted integration motivates considering another dataset for which we know that the

CellPress
OPEN ACCESS

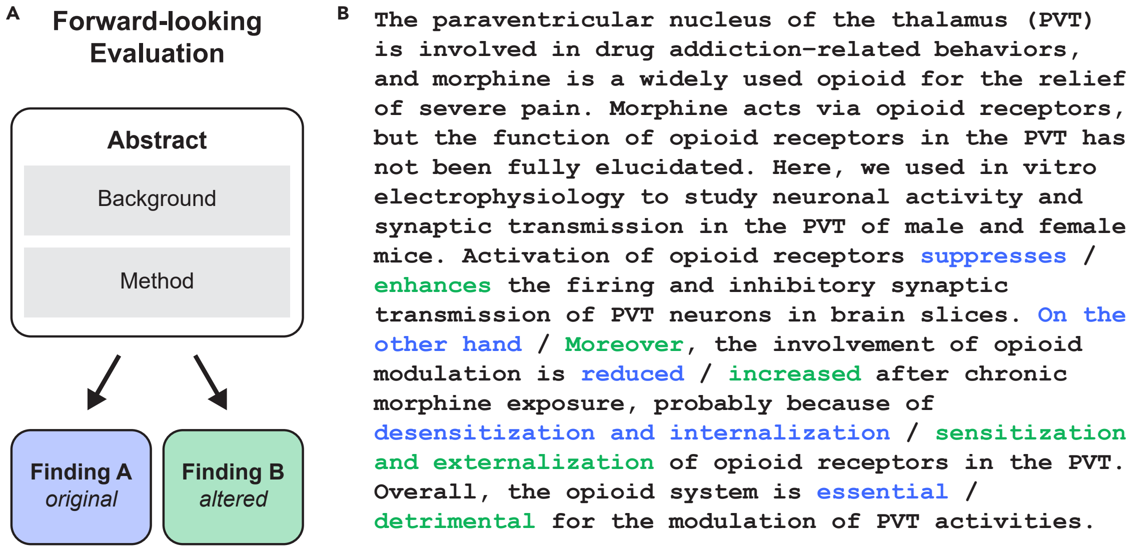

Figure 2. Assessing humans and LLMs using BrainBench

(A) The benchmark comprises test cases constructed from the *Journal of Neuroscience* abstracts. Abstracts consist of background, methods, and results. The test taker chose which of the two versions of the abstract was the original version. The altered version maintained coherence while significantly altering the results. The 100 test cases considered here were constructed by GPT-4 with human oversight and quality control.

(B) An example test case. Humans were instructed to select which version of the abstract was the original by clicking on either the blue or the green text to select that set of options. Test cases varied in the number of alternatives, but a single click chose all options of the same color. After their choice, humans indicated their confidence. LLMs chose the version of the abstract that had the lower perplexity score, and their confidence was assessed by the absolute difference in perplexity of the two options.

confidence of humans and machines is calibrated such that higher confidence is associated with higher accuracy. The next dataset considered provides a valuable test of our standard confidence-weighted model.

Calibration of confidence and classification diversity affords reliable human-LLM teaming

Our contribution also relies on previous efforts that developed BrainBench[14] to assess the capacity of humans and LLMs to predict the outcomes of neuroscience studies. The benchmark includes test cases based on abstracts from the *Journal of Neuroscience*. Each test case contains an original abstract and an altered version (Figure 2). The BrainBench task is to identify the correct study outcome by choosing between the original abstract and its altered counterpart. We evaluated the conditions for effective collaboration (i.e., complementarity), namely, calibration of confidence and classification diversity among team members. Both humans and LLMs were calibrated in that accuracy was positively correlated with confidence (Figure 3A). Diversity held in that LLMs and humans differed on which test items led to errors (Figure 3B). In terms of accuracy (Figure 3C), LLMs numerically surpassed humans by a small margin ($t(2) = 5.20$, $p < 0.05$). Thus, we can consider whether humans can benefit teams consisting of machines that perform comparably or better. Similar to the image classification task, we investigated whether teams including humans performed better than LLM-only teams in the prediction of neuroscience results. All 15 possible team combinations, ranging from individual teammates to a 4-way human-LLM team, were considered (Figure 4). Adding a human teammate to LLM-only teams always improved the team's performance (Welch's $t(8.29) = 8.24$, $p < 0.0001$). Pairing a human with an LLM led to a more effective (i.e., accurate) team than pairing the LLM with a different LLM ($t(2) = 10.39$, $p < 0.01$). In the object recognition task, team performance was not affected by teammates' confidence. Does the fluctuating confidence on a trial-by-trial basis matter for LLMs? Or could complementarity be achieved by just forming a weighted average of responses, as is done in the object recognition task? We evaluated the impact of confidence by setting the magnitude of the confidence scores to 1. This setting mimics the concept of the wisdom of the crowds, where responses are considered without factoring in confidence levels. We find that confidence is particularly important in this knowledge-intensive task, as the overall performance diminishes when confidence scores are neglected (Figure 5). With this variant, human-LLM teams do not always surpass LLM-only teams (Welch's $t(7.36) = 0.38$, $p = 0.36$). The model including confidence (Figure 4) outperforms the variant without confidence (Figure 5) by a significant margin (Welch's $t(17.08) = 3.16$, $p < 0.01$). We then assessed whether our regression approach would benefit from a more elaborate formulation. Equation 4 presents a function that modulates the magnitude of a team member's confidence rating to adjust their calibration. In an optimal setting (Figure S6), the resulting model was indistinguishable from the standard regression model (Welch's $t(19.32) = 0.05$, $p = 0.96$). Adding interaction terms to the confidence-weighted features does not improve the performance of either human-LLM or LLM-only teams (Figure S7). More complex model variants may perform better in other tasks, especially when there are more and less noisy data. Simpler model variants seem to be more robust to noise in our cross-validated

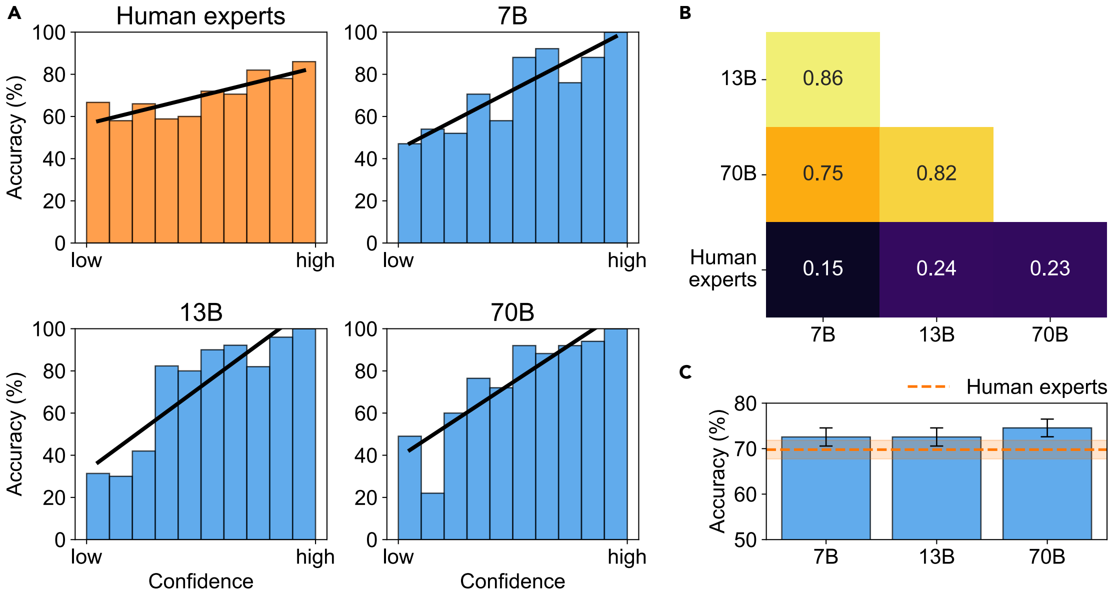

Figure 3. Conditions for effective collaboration between human experts and LLMs are satisfied

(A) When human experts and LLMs were confident in their BrainBench judgments, they were more likely to be correct. Confidence ratings were sorted into equal bins, and the mean accuracy for each bin was plotted. The positive slope of the black regression lines for humans and Llama 2 chat models (7 billion (7B), 13 billion (13B), and 70 billion (70B) parameters) indicates well-calibrated confidence,[14,17–19] meaning higher confidence correlates with higher accuracy.

(B) Item difficulty Spearman correlations among LLMs and human experts. For LLMs, the difference in perplexity between incorrect and correct abstracts was used to determine the relative difficulty of test cases. Mean accuracy was used for human experts. LLMs align more with each other than with humans, which implies human-machine teams will be diverse. The heatmap color scale ranges from 0.1 to 0.9.

(C) LLMs surpass human experts on BrainBench overall. Error bars represent the standard error of the mean using a binomial model.

experiments. Thus, the simplicity of our approach provides effective team collaboration. Our confidence-weighted logistic regression approach follows from the principles of a Bayesian combination model that fosters human-machine complementarity[13] (Figure S1). One question is how well our logistic regression approach compares to the Bayesian approach beyond image classification. Our confidence-weighted regression model outperformed (Figure 6) the Bayesian model when evaluated on the three human-LLM and three LLM-LLM teams for which the Bayesian model is intended to apply (Welch's $t(8.75) = 2.91$, $p < 0.01$). This success is impressive given that the regression approach takes seconds to compute on a current desktop, whereas the Bayesian approach is orders of magnitude slower.

DISCUSSION

Can humans team effectively with machines such as LLMs when the humans perform worse? We developed a confidence-weighted regression approach that can integrate judgments from any number of teammates. Using this method and testing on two forecasting benchmarks,[13,14] we found that human-machine teams achieve complementarity; that is, their combined performance bests that of either teammate alone (Figures 1 and 4). Complementarity was achieved because two critical conditions were satisfied, namely, confidence was well calibrated and classification diversity held among teammates (Figure 3). Strikingly, every combination of machines benefited from adding a human to the team (Figures 1 and 4). Confidence in individual responses significantly impacted team performance in the knowledge-intensive task. The overall performance diminished when confidence scores were neglected (Figure 5). However, in the noisy object recognition task, this effect was not present (Figure S4). In both scenarios, the performance of the confidence-weighted integration of judgments from teammates was equal to or better than the weighted average of responses, i.e., the wisdom of the crowds. For the confidence-weighted model variants to thrive, confidence judgments need to be calibrated such that higher confidence is associated with higher accuracy. Our approach was informed by a Bayesian method for combining judgments of humans and machines.[13] Our approach has a number of advantages, including ease of implementation, very fast runtime, an interpretable solution, and being readily extendable to any number of teammates. Surprisingly, our confidence-weighted regression approach performed better than the Bayesian approach (Figure 6). One possibility is that the discretization of continuous confidence measures, which the Bayesian model requires, limited its performance. Perhaps an alternative formulation would perform better. Unfortunately, reformulating the Bayesian model and properly implementing it requires substantial effort and expertise. In contrast, because our confidence-weighted integration model is formulated within a regression framework, it is straightforward to extend the formulation, for example, by including a function that modulates the calibration of a teammate (Figure S6) or nonlinear relationships (e.g., polynomial terms) between confidence-weighted predictions and outcomes (Figure S7). While we selected three LLMs with superhuman performance on BrainBench, these LLMs are not the highest-performing models on this benchmark.[14] Our

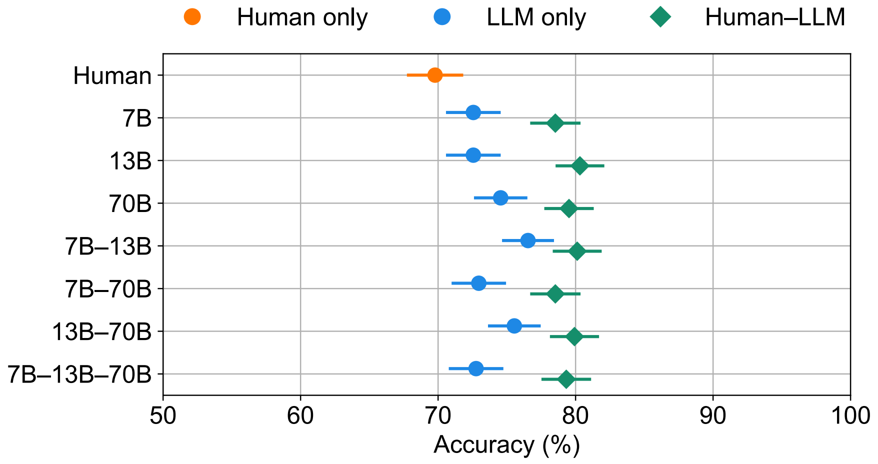

Figure 4. Performance of all possible teams using the confidence-weighted logistic combination model

Adding a human to a team with one or more machines (blue points) always has a benefit (green points). Llama 2 chat models with 7 billion (7B), 13 billion (13B), and 70 billion (70B) parameters are considered. Each data point corresponds to the average across 503 test-case evaluations. Error bars represent the standard error of the mean using a binomial model.

choice was deliberate because a vastly superior teammate may hinder complementarity. In the limit, a teammate who is never wrong does not need to be part of a team. This limiting condition may become more prevalent should LLMs continue to improve and, therefore, diminish the benefits of human-LLM teaming. For the foreseeable future, we suspect there will be tasks for which humans and LLMs can effectively team. Moreover, our method for integrating the judgments of teammates is not limited to human-LLM teams. Instead, the method is general and applies to any set of agents (natural or artificial) that can report how confident they are in their decisions. This study explored the possibility of a collaborative approach between humans and machines for superior decision-making in classifying noisy natural images and forecasting neuroscience outcomes. Our confidence-weighted regression method effectively combined human and machine judgments because teammates fulfilled the conditions of well-calibrated confidence and classification diversity. Our results suggest that there is a place for humans in teams with machines, even when the machines perform better. We hope our work facilitates successful collaborations between humans and machines in addressing important challenges.

METHODS

Datasets

ImageNet 16H

A subset of the 2012 large-scale visual recognition challenge (LSVRC) ImageNet training set[20] was utilized. Namely, the dataset comprised 1,200 test cases (i.e., images) divided equally into 16 classes (chair, oven, knife, bottle, keyboard, clock, boat, bicycle, airplane, truck, car, elephant, bear, dog, cat, and bird). Four levels of phase noise were independently applied to distort the image dataset (Ω = {80,95,110,125}). We considered two noise levels: low (Ω = 80), where humans outperform machines (Figure S2), and high ($\Omega = 125$), where most machines outperform humans (Figure 1). 145 participants classified between 34 and 74 noisy images (i.e., test cases) into the 16 aforementioned categories. For each evaluation, participants also provided a discrete confidence level (low, medium, or high). The total number of human classifications corresponded to 7,247 for low-noise images and 7,239 for high-noise images. Five different machine classifiers pretrained for ImageNet 16H were utilized: AlexNet,[21] DenseNet161,[22] GoogleNet,[23] ResNet152,[24] and VGG-19.[25] One pass through the noisy image data (epoch) was performed during stochastic gradient training. For a given image, the classifiers produced probability scores for each of the 16 classes. The class label was assigned to the class with the highest probability.

BrainBench

The benchmark includes test cases created either by expert neuroscientists or by prompting GPT-4 (Azure OpenAI API; v.2023-05-15) to create test cases. Since LLMs outperform humans by a large margin in both scenarios[14] and substantial differences in performance may preclude complementarity,[13] we used the GPT-4-generated test cases because the performance difference between humans and LLMs was smaller for these test cases, though LLMs were still clearly superior. We considered a dataset comprising 100 machine-generated test cases. These test cases were created from abstracts in the *Journal of Neuroscience* published in 2023. These abstracts are categorized into five sections: behavioral/cognitive, systems/circuits, development/plasticity/repair, neurobiology of disease, and cellular/molecular. Each test case contains a published abstract and an altered version produced by GPT-4. These modifications, though minimal, significantly change the results—for instance, by changing the roles of brain regions or reversing a result's direction (e.g., from "decreases" to "increases"). The altered abstracts remain logically coherent despite the changes. The BrainBench task is to identify the correct study outcome by choosing between the original abstract and its altered counterpart. 171 neuroscience experts were recruited to complete an online study.[14] Each participant evaluated three out of the 100 test cases. Two versions of an abstract were presented: one with the actual results and one that was altered (Figure 2). Participants chose the version they believed to be the original and rated their confidence using a slider bar. After applying several exclusion criteria, the 171 participants yielded 503 observations (2–9 instances per test case). We considered LLMs from the Llama 2 chat family with 7 billion (7B), 13 billion (13B), and 70 billion (70B) parameters.[26] LLMs chose the version of the abstract with the lower perplexity (PPL). Confidence was calculated as the absolute difference in PPL between the original and altered versions of the abstract (Figure 2).

Bayesian combination model

Human and machine judgments were combined by adapting a Bayesian framework for human-machine complementarity.[13]

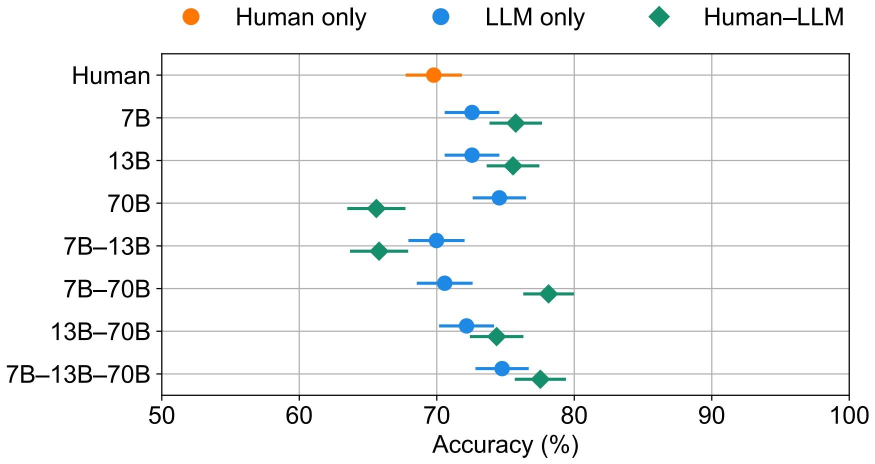

Figure 5. Removing confidence from the logistic combination model diminishes team performance
Accuracy results on the neuroscience forecasting task with the confidence-weighted logistic combination model, where the magnitude of the confidence scores was set to 1, i.e., $f(x) = 1$ in Equation 4. Adding a human to a team with one or more machines (blue points) does not necessarily improve performance (green points). Llama 2 chat models with 7 billion (7B), 13 billion (13B), and 70 billion (70B) parameters are considered. Each data point corresponds to the average across 503 test-case evaluations. Error bars represent the standard error of the mean using a binomial model.

The problem setting for combining two team members, human and machine, is as follows: let N denote the number of test cases to be analyzed with L possible choices. The ground-truth labels of the original test cases are $z \in \{0, \ldots, L-1\}^N$. For the human classifier, the predicted labels $y \in \{0, \ldots, L-1\}^N$ and their corresponding confidence ratings $r \in \{0, \ldots, R-1\}^N$, with "0: lowest possible confidence" and "$R-1$: highest possible confidence," are given. For the machine classifier, we used the probability scores $\pi \in \mathbb{R}_+^{N \times L}$. In the case of LLMs, $\pi = \text{Softmax}(-q)$, where q is the PPL score, reflecting a measure of uncertainty.[14] The first step of this model is to generate correlated probability scores for human and machine classifiers using a bivariate normal distribution:

$$\begin{pmatrix} \pi_H \\ \pi_M \end{pmatrix} \sim \mathcal{N}\left(\begin{pmatrix} \mu_H \\ \mu_M \end{pmatrix}, \begin{pmatrix} \sigma_H^2 & \sigma_H \sigma_M \rho_{HM} \\ \sigma_H \sigma_M \rho_{HM} & \sigma_M^2 \end{pmatrix} \right). \quad \text{(Equation 1)}$$

The means of the underlying distribution, μ_H and μ_M, depend whether the label of test case i, z_i, is correct or not, i.e.,

$$\mu_{i,j,H} = b_H + (a_H - b_H) \cdot 1_{\mathcal{Z}_i}(j) \text{ or}$$

$$\mu_{i,j,M} = b_M + (a_M - b_M) \cdot 1_{\mathcal{Z}_i}(j),$$

with $\mathcal{Z}_i = \{x \mid x = z_i\}$. Note that the scalar parameters a_H, a_M, b_H, b_M, σ_H, σ_M, and ρ_{HM} in Equation 1 are learned from data. The parameter ρ_{HM} from the covariance matrix learns the correlation between the human and machine classifiers. In the case of the machine classifier, π_M is compared to the empirical probability scores, π. Then, for the human classifier, π_H is a latent variable that is used to calculate classifications,

$$y_H \sim \text{Categorical}(\text{Softmax}(\pi_H / \tau)),$$

where τ denotes a temperature parameter, usually small, that helps convergence.[13] The predicted classifications, y_H, are compared to actual human predictions, y. Ordered logit and probit models[27] yield practically indistinguishable results.[28] Departing from Steyver et al.,[13] we used ordered logit because of software availability. We successfully reproduced their prior results with probit using ordered logit (see Figure S1). Ordered logit maps the continuous probability scores, π_H, to an ordinal confidence rating, r_H. This means that

$$r_H \sim \text{OrderedLogistic}(\pi_H, c, \delta), \quad \text{(Equation 2)}$$

where the parameters $c \in \mathbb{R}_+^{R-1}$ are the breakpoints of the intervals that map π_H into r_H and δ is a scalar that controls the sharpness of the rating probability curves. Finally, r_H is compared to the empirical human confidence ratings, r. See the supplemental information for implementation details.

Confidence-weighted logistic combination model

We introduce a logistic regression approach that combines the judgments of any number of teammates. The logistic combination model follows the principles of the Bayesian combination model but is formulated within an easier-to-implement-and-extend regression framework. In its most basic form, which we consider here, each teammate is captured by a single predictor in the regression model. The value of the predictor on a trial depends on the teammate's choice and their confidence. In particular, the magnitude of the predictor is the teammate's confidence on that trial (i.e., confidence-weighted integration), and the sign is determined by the teammate's choice. In general, the fitted β weight for a teammate will reflect their accuracy and calibration. As in the Bayesian combination model, y, r, and π are given. The logistic function is of the form

$$p_x = \frac{1}{1 + e^{-\beta^\top x}},$$

where p_x is the predicted probability of the arbitrarily assigned first option, and the evidence is

$$\beta^\top x = \beta_I + \beta_H x_H + \beta_M x_M. \quad \text{(Equation 3)}$$

The fitted weights β_I, β_H, and β_M correspond to the intercept and human and machine teammates, respectively. The term $x_{i,j,k}$ is the signed confidence of the i-th test case for the class label j selected by team member k. For human participants, $x_{i,j,H}$ is r_i if $y_i = j$ is the selected class label and $-r_i$ otherwise. Similarly, for machine team members, $x_{i,j,M} = \pi_{i,j}$ if $y_i = j$ is the selected class label and $x_{i,j,M} = -\pi_{i,j}$ otherwise. In the binary case of LLMs, we consider the absolute PPL difference as a measure of confidence. This means that $x_{i,0,M}$ is $|\Delta q_i|$ if $y_i = 0$ is the selected class label and $-|\Delta q_i|$ otherwise. Consider the case where an agent is

CellPress
OPEN ACCESS

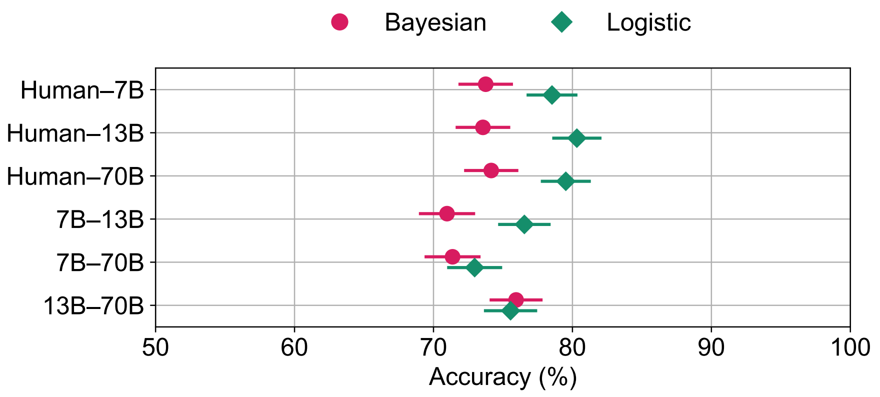

Figure 6. Comparison between Bayesian and confidence-weighted logistic combination models for human-LLM and LLM-LLM teams

The confidence-weighted logistic combination model more effectively integrates human and machine judgments. Llama 2 chat models with 7 billion (7B), 13 billion (13B), and 70 billion (70B) parameters are considered. Each data point corresponds to the average across 503 test-case evaluations. Error bars represent the standard error of the mean using a binomial model.

accurate but its confidence is not calibrated. Then, a slightly more complex version of this logistic regression can be formulated by introducing a single-parameter function for each team member. This function can either pass through the magnitude of a team member's confidence rating or squash its magnitude toward 1 for every confidence rating, e.g.,

$$f(x) = 1 + \frac{x - 1}{1 + \alpha|x - 1|}. \quad \text{(Equation 4)}$$

That more complex model should work better if an agent is a good predictor of outcomes but its confidence is random. In that case, α will be high, and the magnitude of the confidence weighting will always be $f(x) = 1$ in Equation 4. Thus, the fitted weights for logistic regression would not penalize the agent as much in the combination. For a really well-calibrated agent, α should be close to 0. Thus, Equation 4 becomes the identity function, $f(x) = x$. It turns out that for the two tasks, there was no significant difference when including a more complex model optimizing α. However, the no-confidence variant (Figure 5) was considered as a foil to our approach, which values confidence. Additionally, this model can be easily expanded by including additional terms to Equation 3. For example, a third fitted weight could be included for an interaction term $x_H x_M$. Likewise, polynomial regression could be used to include x_H^2 and x_M^2 and corresponding fitted weights.

Cross-validation procedure

Given a total of M observations across N different test cases (with $M > N$), we performed a leave-one-out cross-validation (LOOCV) that provides the best bias-variance trade-off for small datasets. We evaluated the consistency of performance estimates across different cross-validation procedures, demonstrating that our results reflect true model generalization rather than artifacts of a particular validation strategy (Figure S8). Consider the evaluation of the i-th test case. With this procedure, we removed all instances of test case i, leaving the remaining $N - 1$ test cases with all their instances to train the classifier teams. For testing, we utilized all the instances of test case i. This was repeated for all N test cases, yielding M predictions. Note that for individual teammates, the evaluation comprised only the testing phase. When the predicted labels of any team or individual teammate in this study were randomly shuffled, the LOOCV accuracy dropped to approximately chance level (i.e., $1/L$).

RESOURCE AVAILABILITY

Lead contact

Requests for further information and resources should be directed to and will be fulfilled by the lead contact, Felipe Yáñez (felipe.yanez@mpinb.mpg.de).

Materials availability

This study did not generate new unique reagents.

Data and code availability

For ImageNet 16H[13] and BrainBench,[14] the previously reported human participant data and machine confidence scores utilized in each study are available at https://osf.io/2ntrf and https://github.com/braingpt-lovelab/BrainBench, respectively. All computer code associated with this work, including combination model implementations, team evaluations, and analyses, is publicly available at https://github.com/braingpt-lovelab/haico and has been archived in Edmond.[29]

ACKNOWLEDGMENTS

This work was supported by a NeuroData Discovery Award from the Kavli Foundation to F.Y., the ESRC (ES/W007347/1), Microsoft (Accelerate Foundation Models Research Program), a Royal Society Wolfson Fellowship (18302), an AI safety grant from the Foresight Institute, and the Laboratory Directed Research and Development program of Los Alamos National Laboratory under project number 20250637DI to B.C.L.

AUTHOR CONTRIBUTIONS

Conceptualization, F.Y. and B.C.L.; methodology, F.Y., X.L., and B.C.L.; investigation, F.Y. and X.L.; writing – original draft, F.Y.; writing – review & editing, F.Y., X.L., and B.C.L.; funding acquisition, F.Y. and B.C.L.; resources, F.Y. and O.V.M.; supervision, B.C.L.

DECLARATION OF INTERESTS

The authors declare no competing interests.

SUPPLEMENTAL INFORMATION

Supplemental information can be found online at https://doi.org/10.1016/j.patter.2025.101423.

Received: March 24, 2025
Revised: September 15, 2025
Accepted: October 20, 2025
Published: November 20, 2025

REFERENCES

1. Eppler, M.J., and Mengis, J. (2004). The Concept of Information Overload: A Review of Literature from Organization Science, Accounting, Marketing, MIS, and Related Disciplines. Inf. Soc. *20*, 325–344. https://doi.org/10.1080/01972240490507974.
2. Bawden, D., and Robinson, L. (2009). The dark side of information: overload, anxiety and other paradoxes and pathologies. J. Inf. Sci. *35*, 180–191. https://doi.org/10.1177/0165551508095781.
3. LeCun, Y., Bengio, Y., and Hinton, G. (2015). Deep learning. Nature *521*, 436–444. https://doi.org/10.1038/nature14539.
4. Silver, D., Huang, A., Maddison, C.J., Guez, A., Sifre, L., van den Driessche, G., Schrittwieser, J., Antonoglou, I., Panneershelvam, V., Lanctot, M., et al. (2016). Mastering the game of Go with deep neural networks and tree search. Nature *529*, 484–489. https://doi.org/10.1038/nature16961.
5. Jumper, J., Evans, R., Pritzel, A., Green, T., Figurnov, M., Ronneberger, O., Tunyasuvunakool, K., Bates, R., Žídek, A., Potapenko, A., et al. (2021). Highly accurate protein structure prediction with AlphaFold. Nature *596*, 583–589. https://doi.org/10.1038/s41586-021-03819-2.
6. Zhang, T., Ladhak, F., Durmus, E., Liang, P., McKeown, K., and Hashimoto, T.B. (2024). Benchmarking Large Language Models for News Summarization. Trans. Assoc. Comput. Linguist. *12*, 39–57. https://doi.org/10.1162/tacl_a_00632.
7. Radford A., Wu J., Child R., Luan D., Amodei D., Sutskever I. (2019). Language Models are Unsupervised Multitask Learners. OpenAI blog. https://openai.com/index/better-language-models/.
8. Brown, T., Mann, B., Ryder, N., Subbiah, M., Kaplan, J.D., Dhariwal, P., Neelakantan, A., Shyam, P., Sastry, G., Askell, A., et al. (2020). Language Models are Few-Shot Learners. In Advances in Neural Information Processing Systems, *33*, H. Larochelle, M. Ranzato, R. Hadsell, M. Balcan, and H. Lin, eds.*, pp. 1877–1901*.
9. Brynjolfsson, E., and McAfee, A. (2014). The Second Machine Age: Work, Progress, and Prosperity in a Time of Brilliant Technologies (New York, NY, USA: W. W. Norton & Company).
10. Frey, C.B., and Osborne, M.A. (2017). The future of employment: How susceptible are jobs to computerisation? Technol. Forecast. Soc. Change *114*, 254–280. https://doi.org/10.1016/j.techfore.2016.08.019.
11. Vaccaro, M., Almaatouq, A., and Malone, T. (2024). When combinations of humans and AI are useful: A systematic review and meta-analysis. Nat. Hum. Behav. *8*, 2293–2303. https://doi.org/10.1038/s41562-024-02024-1.
12. Hemmer, P., Schemmer, M., Kühl, N., Vössing, M., and Satzger, G. (2025). Complementarity in human-ai collaboration: concept, sources, and evidence. Eur. J. Inf. Syst. 1–24. https://doi.org/10.1080/0960085X.2025.2475962.
13. Steyvers, M., Tejeda, H., Kerrigan, G., and Smyth, P. (2022). Bayesian modeling of human–AI complementarity. Proc. Natl. Acad. Sci. USA *119*, e2111547119. https://doi.org/10.1073/pnas.2111547119.
14. Luo, X., Rechardt, A., Sun, G., Nejad, K.K., Yáñez, F., Yilmaz, B., Lee, K., Cohen, A.O., Borghesani, V., Pashkov, A., et al. (2025). Large language models surpass human experts in predicting neuroscience results. Nat. Hum. Behav. *9*, 305–315. https://doi.org/10.1038/s41562-024-02046-9.
15. Steyvers, M., Tejeda, H., Kumar, A., Belem, C., Karny, S., Hu, X., Mayer, L.W., and Smyth, P. (2025). What large language models know and what people think they know. Nat. Mach. Intell. *7*, 221–231. https://doi.org/10.1038/s42256-024-00976-7.
16. Geirhos, R., Temme, C.R.M., Rauber, J., Schütt, H.H., Bethge, M., and Wichmann, F.A. (2018). Generalisation in humans and deep neural networks. In Advances in Neural Information Processing Systems, *31*, S. Bengio, H. Wallach, H. Larochelle, K. Grauman, N. Cesa-Bianchi, and R. Garnett, eds.*, pp. 1–13*.
17. Keren, G. (1991). Calibration and probability judgements: Conceptual and methodological issues. Acta Psychol. *77*, 217–273. https://doi.org/10.1016/0001-6918(91)90036-Y.
18. Baranski, J.V., and Petrusic, W.M. (1994). The calibration and resolution of confidence in perceptual judgments. Percept. Psychophys. *55*, 412–428. https://doi.org/10.3758/BF03205299.
19. Tian, K., Mitchell, E., Zhou, A., Sharma, A., Rafailov, R., Yao, H., Finn, C., and Manning, C. (2023). Just Ask for Calibration: Strategies for Eliciting Calibrated Confidence Scores from Language Models Fine-Tuned with Human Feedback. In Proceedings of the Conference on Empirical Methods in Natural Language Processing, H. Bouamor, J. Pino, and K. Bali, eds. (Association for Computational Linguistics), pp. 5433–5442. https://doi.org/10.18653/v1/2023.emnlp-main.330.
20. Russakovsky, O., Deng, J., Su, H., Krause, J., Satheesh, S., Ma, S., Huang, Z., Karpathy, A., Khosla, A., Bernstein, M., et al. (2015). ImageNet Large Scale Visual Recognition Challenge. Int. J. Comput. Vis. *115*, 211–252. https://doi.org/10.1007/s11263-015-0816-y.
21. Krizhevsky, A., Sutskever, I., and Hinton, G.E. (2012). ImageNet Classification with Deep Convolutional Neural Networks. In Advances in Neural Information Processing Systems, *25*, F. Pereira, C. Burges, L. Bottou, and K. Weinberger, eds.*, pp. 1–9*.
22. Huang, G., Liu, Z., van der Maaten, L., and Weinberger, K.Q. (2017). Densely Connected Convolutional Networks. In Proceedings of the IEEE Conference on Computer Vision and Pattern Recognition (CVPR) (IEEE), pp. 4700–4708. https://doi.org/10.1109/CVPR.2017.243.
23. Szegedy, C., Liu, W., Jia, Y., Sermanet, P., Reed, S., Anguelov, D., Erhan, D., Vanhoucke, V., and Rabinovich, A. (2015). Going Deeper With Convolutions. In Proceedings of the IEEE Conference on Computer Vision and Pattern Recognition (CVPR) (IEEE), pp. 1–9. https://doi.org/10.1109/CVPR.2015.7298594.
24. He, K., Zhang, X., Ren, S., and Sun, J. (2016). Deep Residual Learning for Image Recognition. In Proceedings of the IEEE Conference on Computer Vision and Pattern Recognition (CVPR) (IEEE), pp. 770–778. https://doi.org/10.1109/CVPR.2016.90.
25. Simonyan, K., and Zisserman, A. (2015). Very Deep Convolutional Networks for Large-Scale Image Recognition. Preprint at arXiv. https://doi.org/10.48550/arXiv.1409.1556.
26. Touvron, H., Martin, L., Stone, K., Albert, P., Almahairi, A., Babaei, Y., Bashlykov, N., Batra, S., Bhargava, P., Bhosale, S., et al. (2023). Llama 2: Open Foundation and Fine-Tuned Chat Models. Preprint at arXiv. https://doi.org/10.48550/arXiv.2307.09288.
27. McCullagh, P. (1980). Regression Models for Ordinal Data. J. Roy. Stat. Soc. B *42*, 109–127. https://doi.org/10.1111/j.2517-6161.1980.tb01109.x.
28. Boes, S., and Winkelmann, R. (2006). Ordered response models. All. Stat. Arch. *90*, 167–181. https://doi.org/10.1007/s10182-006-0228-y.
29. Yáñez, F., Luo, X., Valerio Minero, O., and Love, B.C. (2025). Source code for ‘‘Confidence-weighted integration of human and machine judgments for superior decision-making’’. Edmond. https://doi.org/10.17617/3.IGVPQV.

Patterns, Volume *7*

Supplemental information

Confidence-weighted integration of human and machine judgments for superior decision-making

Felipe Yáñez, Xiaoliang Luo, Omar Valerio Minero, and Bradley C. Love

SUPPLEMENTAL FIGURES

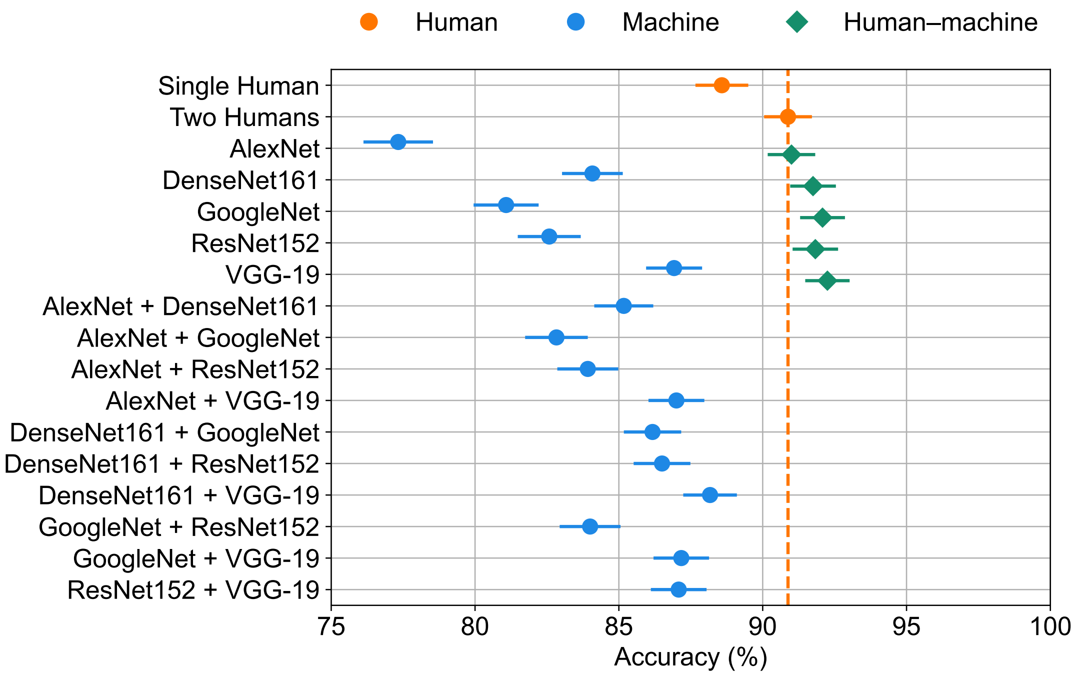

Figure S1. Performance of the Bayesian combination models in the low-noise object recognition task [S1]

We reproduced Steyver's et al.'s [S1] results using ordered logit (see Equation 2), as opposed to probit. Accuracy results on low levels of image noise ($\Omega = 80$) with the Bayesian combination model, reproducing the original setting. We used 1200 predictions (corresponding to 1200 unique images) and split them into four random partitions for the purpose of four-fold cross-validation. Two human participants were created by random sampling without replacement across the 1200 unique images. In this case, machine classifiers are surpassed by humans ($t(4) = -3.88$, $P < 0.01$). Teams comprising a human and a machine (green points) deliver superior results compared to machine-only and machine–machine teams (Welch's $t(16.04) = 9.18$, $P < 0.0001$). Each data point corresponds to the average across 1200 image evaluations. Error bars represent standard error of the mean using a binomial model.

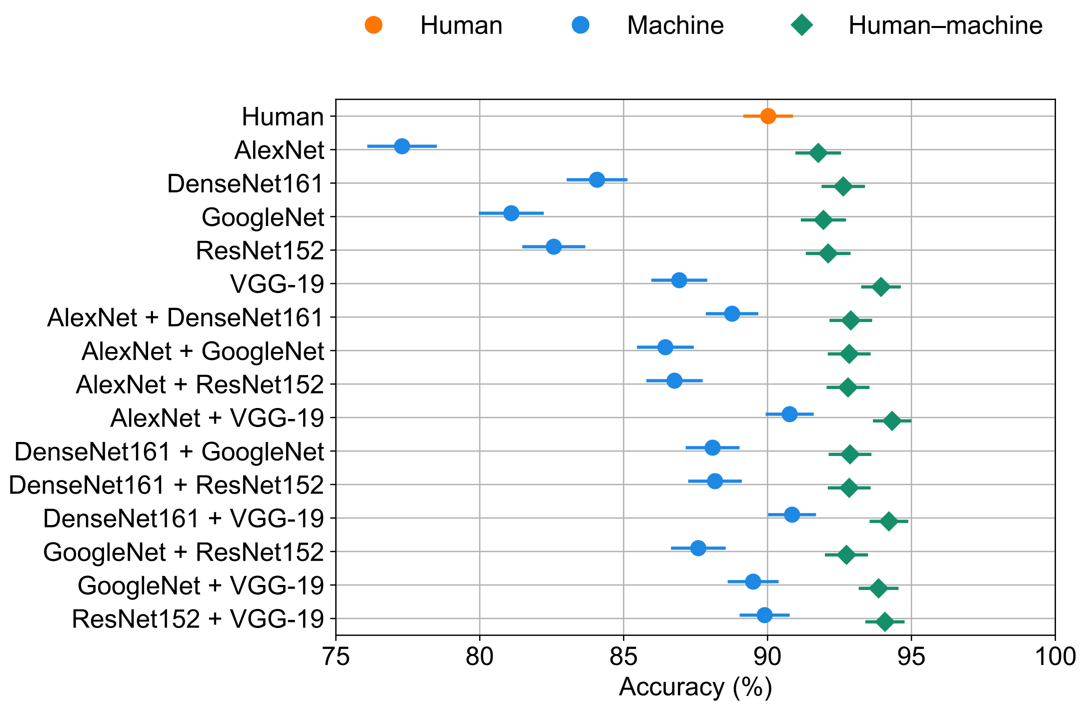

Figure S2. Performance of the confidence-weighted logistic combination model in the low-noise object recognition task [S1]

Accuracy results on low levels of image noise ($\Omega = 80$) with the confidence-weighted combination model, where humans outperform machines ($t(4) = -4.77$, $P < 0.01$). Human–machine teams consistently outperform teams with one or more machines (Welch's $t(37.46) = 12.50$, $P < 0.0001$). Each data point corresponds to the average across 7247 image evaluations. Error bars represent standard error of the mean using a binomial model.

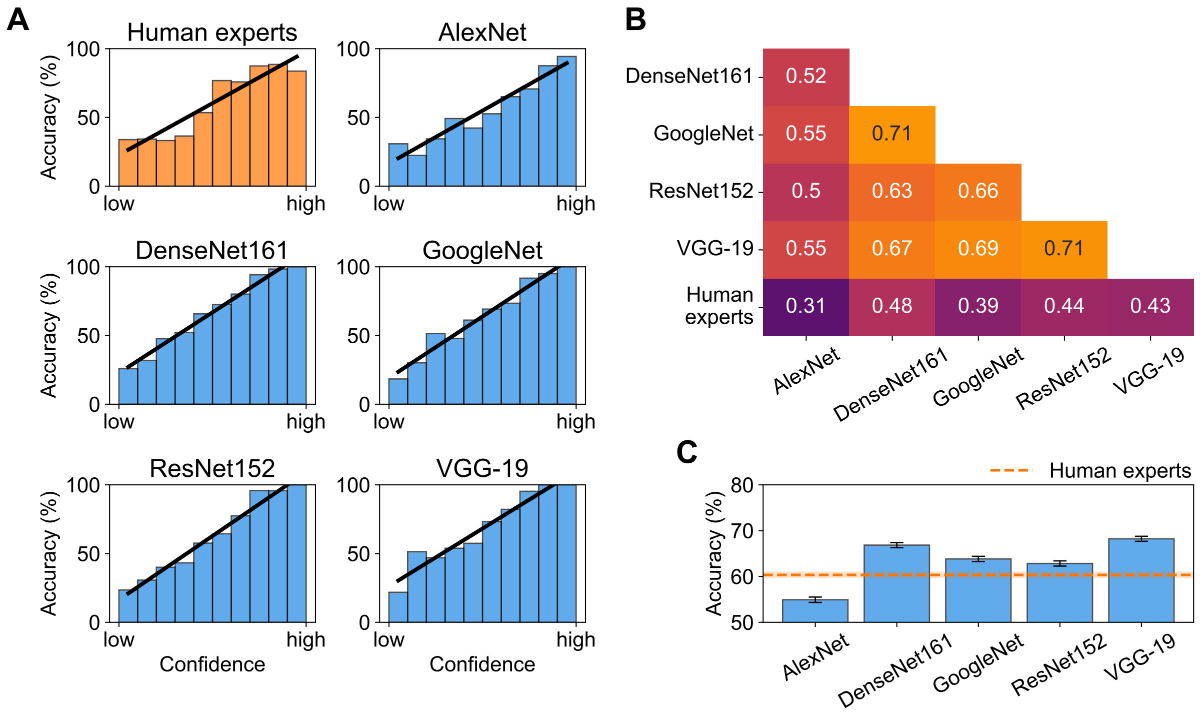

Figure S3. Conditions for effective collaboration between human experts and machines are satisfied for the noisy object recognition task [S1]

(A) When human experts and machines were confident in their judgments, they were more likely to be correct. Confidence ratings were sorted into equal bins, and the mean accuracy for each bin was plotted. The positive slope of the black regression lines for humans and models (AlexNet, DenseNet161, GoogleNet, ResNet152, and VGG-19) indicates well-calibrated confidence [S2–S5], meaning higher confidence correlates with higher accuracy.

(B) Item difficulty Spearman correlations among machines and human experts. For machines, we used the probability score of the predicted image class to determine relative image classification difficulty. The probability score was then signed with $+1$ if the prediction was correct and -1 if it was incorrect. Mean accuracy was used for human experts. Heatmap color scale ranges from 0.1 to 0.9.

(C) Besides AlexNet, models surpass human experts on this task. Error bars represent standard error of the mean using a binomial model.

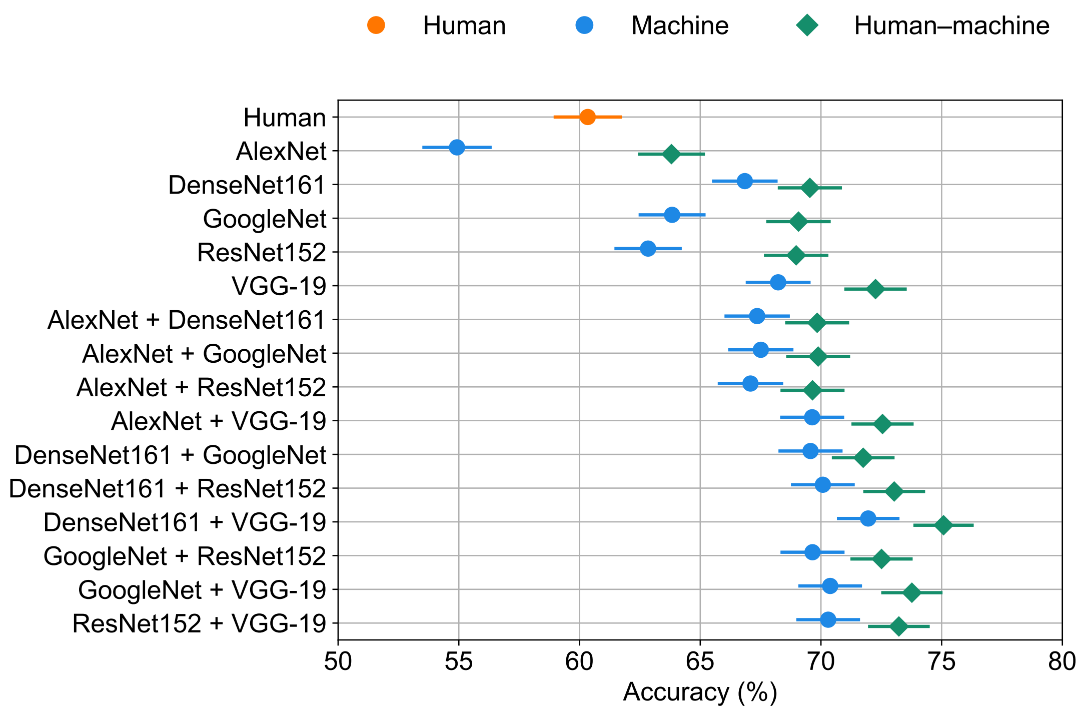

Figure S4. Removing confidence from the confidence-weighted logistic combination model does not negatively impact team performance in the noisy object recognition task [S1]

Accuracy results on high levels of image noise ($\Omega = 125$) with the confidence-weighted logistic combination model, where the probability scores were set to 1, i.e., $f(x) = 1$ in Equation 4. Similarly as in the base scenario (Figure 1), human–machine teams surpass machine-only teams (Welch's $t(24.08) = 2.80$, $P < 0.01$), although with a smaller margin. Each data point corresponds to the average across 7239 image evaluations. Error bars represent standard error of the mean using a binomial model.

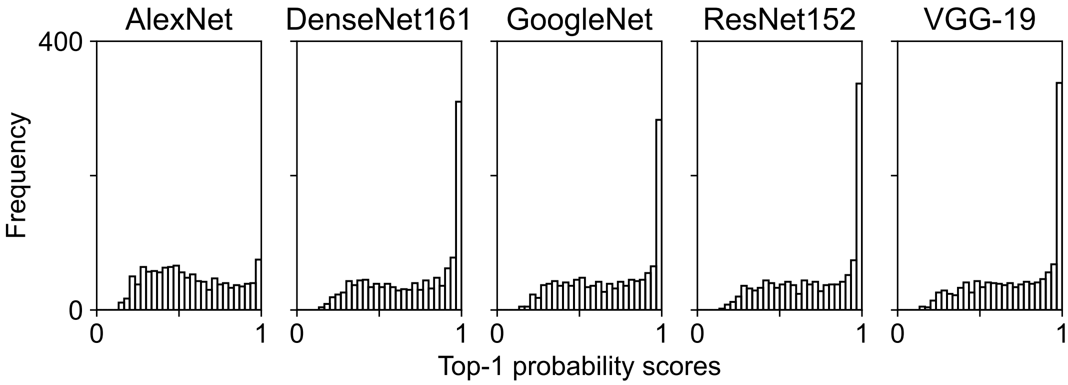

Figure S5. Confidence assessment in the noisy object recognition task [S1]

Histogram of top-1 probability scores under high image noise ($\Omega = 125$) for different machine classifiers. For each test case ($n = 1200$), only the highest predicted probability was used, and values were grouped into 30 bins. The resulting distributions are heavily skewed toward 1. To test whether confidence magnitudes improve predictions, we used cross-entropy, a metric that reflects both accuracy and calibration. For each classifier, cross-entropy was computed independently for both signed probability scores (i.e., signed confidence) and sign-only features. The results are nearly identical (Welch's $t(7.99) = 0.24$, $P = 0.82$), suggesting that confidence magnitudes provide no additional benefit in this setting.

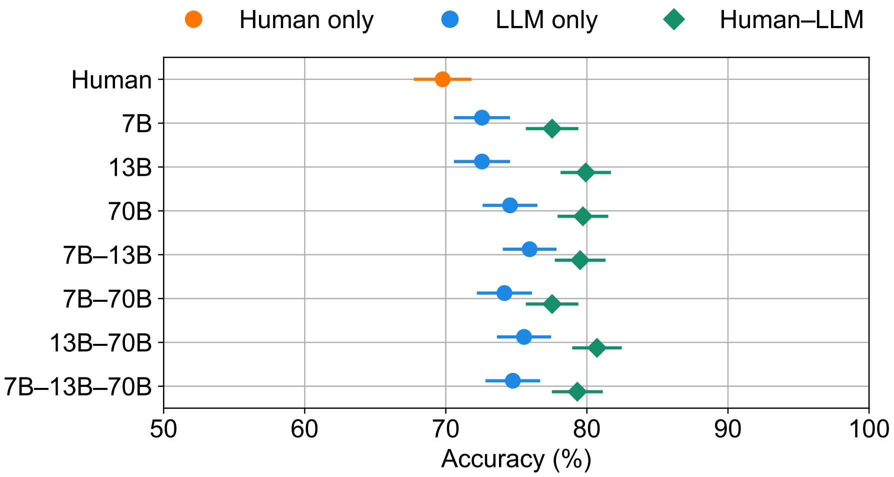

Figure S6. Optimal squashing in the confidence-weighted logistic combination model does not improve overall team performance in the neuroscience forecasting task [S5]

Accuracy results on the neuroscience forecasting task with the confidence-weighted logistic combination model, where the parameter α in Equation 4 was optimized. Similarly as in the base scenario (Figure 4), human–LLM teams surpass LLM-only teams (Welch's $t(11.89) = 7.20$, $P < 0.0001$). The performance between base and squashing scenarios is, however, indistinguishable (Welch's $t(19.32) = 0.05$, $P = 0.96$). Each data point corresponds to the average across 503 test case evaluations. Error bars represent standard error of the mean using a binomial model.

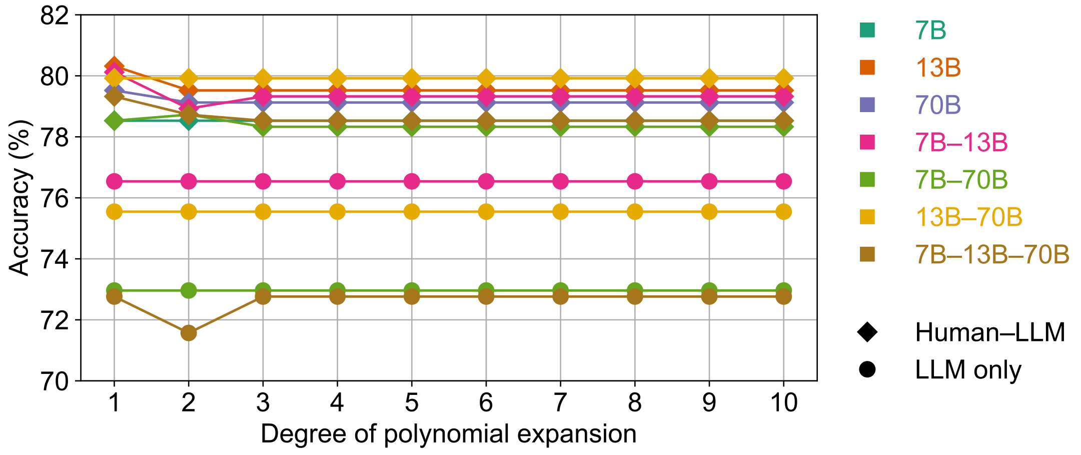

Figure S7. Adding interaction terms to the confidence-weighted logistic combination model does not improve overall team performance in the neuroscience forecasting task [S5]

Accuracy results of the confidence-weighted logistic combination model on the neuroscience forecasting task as a function of the degree of the polynomial expansion of the features. Only interaction terms were considered in the polynomial expansion, as the results including pure terms were identical. Team performance is independent of interaction terms. Each data point corresponds to the average across 503 test case evaluations.

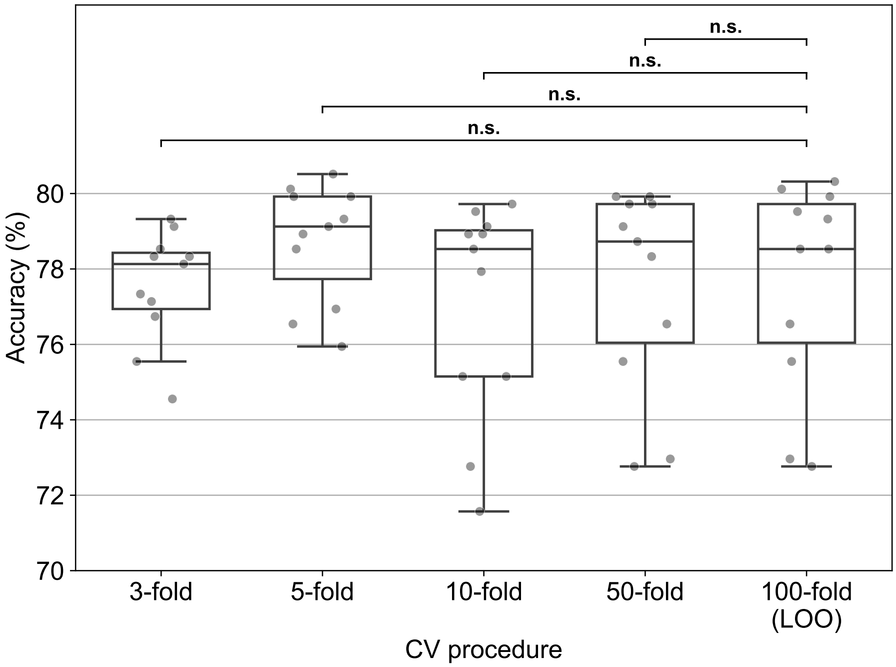

Figure S8. Performance consistency analysis across different cross-validation procedures

Box plots show the distribution of overall accuracy for 3-fold, 5-fold, 10-fold, 50-fold, and leave-one-out (LOO) cross-validation (CV) procedures for the experiment presented in Figure 4. Overlaid data points represent human–machine and machine–machine team predictions. Paired t-tests (parametric) and Wilcoxon signed-rank tests (non-parametric) were used to compare each CV procedure with LOOCV to ensure robust assessment regardless of data distribution assumptions. Results show non-significant differences, with similar mean accuracies (ranging from 77.0% to 78.7%) and overlapping 95% bootstrap confidence intervals (widths between 1.8% and 3.2%). This demonstrates that LOOCV is an appropriate validation procedure to evaluate model performance.

SUPPLEMENTAL METHODS

Implementation of Bayesian Combination Model

We implemented the Bayesian combination model [S1] in Python [S6], which was originally developed in JAGS. The model comprises two stages: parameter inference (training) and class label prediction (testing). Algorithm S1 illustrates the inference procedure for the human–machine case. During testing, the posterior samples of the parameters learned in training are used to evaluate unseen data. For each posterior sample, we compute the joint log-likelihood of the machine scores, human classifications, and human confidence ratings under each possible class. These log-likelihoods are aggregated across samples and normalized via a softmax transformation. The predicted class label corresponds to the class with the highest probability.

In Figure S1, we reproduced the experiment from Figure 3 (Top) in Steyver's et al. [S1] using our Python implementation. The overall team performance ($n = 16$) obtained with our method is indistinguishable from the original JAGS implementation (Welch's $t(28.95) = -0.24$, $P = 0.81$). This also holds for the five human–machine (Welch's $t(5.33) = -2.17$, $P = 0.08$), and ten machine–machine (Welch's $t(17.60) = 0.17$, $P = 0.87$) teams.

Algorithm S1 Bayesian Combination Model

1: Given data: true class labels z, probability scores π_M, human classification y, and confidence ratings r.

2: Set priors: $a_M \sim \mathcal{N}(0, 10)$, $b_M \sim \mathcal{N}(0, 10)$, $\sigma_M \sim$ Uniform$(0, 15)$, $a_H \sim \mathcal{N}(0, 10)$, $b_H = 0$, $\sigma_H = 1$, $\rho \sim$ Uniform$(-1, 1)$, $\tau = 0.05$, $c \sim$ Uniform$(0, 1)$ with $c_i < c_{i+1}\ \forall\, i = 1, \ldots, R-2$, and $\delta \sim$ Uniform$(0, 100)$.

3: **start parameter inference:**

4: $\quad \mu_{i,j,M} \leftarrow b_M + (a_M - b_M) \cdot \mathbf{1}_{\mathcal{Z}_i}(j)$

5: $\quad \mu_{i,j,H} \leftarrow b_H + (a_H - b_H) \cdot \mathbf{1}_{\mathcal{Z}_i}(j)$

6: $\quad \pi_M \sim \mathcal{N}(\mu_M, \sigma_M)$ ▷ Compare to actual data

7: $\quad \pi_H \sim \mathcal{N}\left(\mu_H + \rho\, \sigma_H \left(\frac{\pi_M - \mu_M}{\sigma_M}\right),\ \sqrt{1-\rho^2}\, \sigma_H\right)$

8: $\quad y \sim$ Categorical (Softmax (π_H/τ)) ▷ Compare to actual data

9: $\quad r \sim$ OrderedLogistic(π_H, c, δ) ▷ Compare to actual data

10: **end parameter inference**

We assumed that all human participants shared the same set of parameters (a_H , b_H , σ_H, c, δ, and τ). In the neuroscience forecasting task, human confidence ratings on the slider bar were mapped to range between 1 and 100. A wide range is computationally expensive, thus, we aggregate it into three levels: "0: low confidence", "1: moderate confidence", and "2: high confidence". Then, the aggregated confidence rating used for analysis, $r \in \{0, 1, 2\}^N$, reads

$$r = \begin{cases} 0 & \text{if} \quad \text{self-reported confidence} \le 33, \\ 1 & \text{if} \quad 33 < \text{self-reported confidence} \le 66, \\ 2 & \text{if} \quad 66 < \text{self-reported confidence} \end{cases}.$$

The utilized cutpoints (i.e., 33 and 66) produced a good agreement between confidence and accuracy. Among the evaluations of human participants, "low" had 63.2% average accuracy ($n = 174$), "moderate" had 66.5% ($n = 185$), and "high" had 81.9% ($n = 144$). To infer the posterior over the underlying parameters, a No-U-Turn Sampler (NUTS) for Markov chain Monte Carlo (MCMC) [S7] was used with $n_w = 1000$ warmup steps, $n_c = 8$ chains, and $n_s = 50$ samples.

SUPPLEMENTAL REFERENCES

[S1] Steyvers, M., Tejeda, H., Kerrigan, G., and Smyth, P. (2022). Bayesian modeling of human–AI complementarity. PNAS *119*, e2111547119. https://doi.org/10.1073/pnas.2111547119.

[S2] Keren, G. (1991). Calibration and probability judgements: Conceptual and methodological issues. Acta Psychol. *77*, 217–273. https://doi.org/10.1016/0001-6918(91)90036-Y.

[S3] Baranski, J. V. and Petrusic, W. M. (1994). The calibration and resolution of confidence in perceptual judgments. Percept. Psychophys. *55*, 412–428. https://doi.org/10.3758/BF03205299.

[S4] Tian, K., Mitchell, E., Zhou, A., Sharma, A., Rafailov, R., Yao, H., Finn, C., and Manning, C. (2023). Just Ask for Calibration: Strategies for Eliciting Calibrated Confidence Scores from Language Models Fine-Tuned with Human Feedback. In Proceedings of the 2023 Conference on Empirical Methods in Natural Language Processing, H. Bouamor, J. Pino, and K. Bali, eds. (Association for Computational Linguistics), pp. 5433–5442. https://doi.org/10.18653/v1/2023.emnlp-main.330.

[S5] Luo, X., Rechardt, A., Sun, G., Nejad, K. K., Yáñez, F., Yilmaz, B., Lee, K., Cohen, A. O., Borghesani, V., Pashkov, A., et al. (2025). Large language models surpass human experts in predicting neuroscience results. Nat. Hum. Behav. *9*, 305–315. https://doi.org/10.1038/s41562-024-02046-9.

[S6] Yáñez, F., Luo, X., Valerio Minero, O., and Love, B. C. (2025). Source code for "Confidence-weighted integration of human and machine judgments for superior decision-making". Edmond. https://doi.org/10.17617/3.IGVPQV.

[S7] Bingham, E., Chen, J. P., Jankowiak, M., Obermeyer, F., Pradhan, N., Karaletsos, T., Singh, R., Szerlip, P., Horsfall, P., and Goodman, N. D. (2019). Pyro: Deep Universal Probabilistic Programming. J. Mach. Learn. Res. *20*, 1–6. http://jmlr.org/papers/v20/18-403.html.
